# Utilizing Baidu Index to Track Online Interest in Influenza During the COVID-19 Pandemic in China

**DOI:** 10.7759/cureus.27582

**Published:** 2022-08-01

**Authors:** Ziying He, Luyan Teng, Qinyi Tan

**Affiliations:** 1 School of Geographical Sciences, Southwest University, Chongqing, CHN; 2 College of International Education, Sichuan International Studies University, Chongqing, CHN; 3 Center for Studies of Education and Psychology of Ethnic Minorities in Southwest China, Southwest University, Chongqing, CHN

**Keywords:** china, baidu index, covid-19 pandemic, big data, influenza

## Abstract

Background

Influenza is commonly called the flu which is a contagious respiratory illness caused by influenza viruses that infect the nose, throat, and sometimes the lungs, usually a self-limiting, febrile disease of global importance. It occurs every year and infects the respiratory tract and can lead to sporadic, local outbreaks of widespread epidemics. The global burden of influenza epidemics on incidence rate and mortality is considerable. It is noted that patients with early coronavirus disease-2019 (COVID-19) have symptoms such as headache, nasal congestion, sneezing, and cough, which are like those of influenza. And the outbreak of COVID-19 coincided with the winter and spring season in the northern hemisphere with a high incidence of influenza. And it leads to the public's attention to influenza.

Method

In order to better clarify the social concern of Chinese people about "influenza" during the COVID-19 pandemic, this study conducted a trends analysis using the Baidu index from January 1, 2018, to January 1, 2022, and compared the public's search index with "COVID-19" during this period. This study used ArcGIS version 10.4 (https://www.esri.com/) to conduct a Global Moran's I analysis of the public concern of "influenza" in 31 provinces (municipalities directly under the central government and autonomous regions) in China from 2018 to 2021, except for Hong Kong, Macao, and Taiwan and a Local Moran's I of the "influenza" concern in 2018 and 2021.

Results

We observed that before the outbreak of COVID-19, the search trend of the public for "influenza" was concentrated in the winter and spring of each year, showing seasonal characteristics. However, after the outbreak of COVID-19, the public's search trend for "influenza" increased sharply, and then it leveled off. This shows completely that there is a certain correlation between the COVID-19 outbreak and the online search for "influenza". Regarding the Global Moran's I, the spatial clustering of national "influenza" concerns was observed.

During the COVID-19 pandemic, the spatial correlation between the magnitude of public concern and the spatial correlation became larger as the number of years increased and is greater than that before the outbreak of the COVID-19 pandemic. The results of Local Moran's I showed that the main types of local spatial autocorrelation in 2018 and 2021 were both positive high-high correlations, but the former was mainly concentrated in the eastern coastal region, while the latter began to spread to the central region.

Conclusion

The analysis of the Baidu Index shows that during the COVID-19 pandemic, the public's interest in "influenza" first increased and then decreased, and then remained at a trough, no longer showing the seasonal change characteristics before the outbreak of the COVID-19, indicating that there may be a correlation between COVID-19 and "influenza". The Moran's I indicate that the national "influenza" concern is spatially clustered, while the spatial correlation is increasing and greater than before the outbreak of the COVID-19 pandemic. This is most likely related to the daily update of information related to patients with COVID-19. Meanwhile, the "high-high" local clustering of "influenza" concerns in the central and eastern regions during the COVID-19 pandemic is related to the frequent human and logistic exchanges in the central and eastern regions, which contributed to the spread of the disease.

## Introduction

The most serious epidemic known is the influenza pandemic from 1918 to 1919, which caused an estimated 20 million deaths worldwide [1]. The influenza virus spreads around the world and can affect all age groups, causing serious public health problems [2]. It is reported that the Asian flu in 1957 and the Hong Kong flu in 1968 killed about 1 million people [3]. The clinical picture of the novel coronavirus disease-2019 (COVID-19) shows significant similarity with influenza [4]. Influenza and COVID-19 are both contagious respiratory illnesses, but they are caused by different viruses. The COVID-19 pandemic is caused by a novel virus severe acute respiratory syndrome coronavirus 2 (SARS-CoV-2). Influenza is an infectious respiratory disease, caused by influenza A and influenza B viruses [5].

With the passage of time, new influenza virus subtypes are emerging due to the gradual variation or drift of antigens [6]. According to the data from the World Health Organization, as of January 2, 2022, the COVID-19 pandemic has infected nearly 289 million people and has caused more than 5.4 million deaths worldwide. The symptoms of patients with COVID-19 are like those of patients with influenza, including fever, headache, muscle ache, and so on [7-11]. The COVID-19 outbreak occurred in the winter of 2019 [4], which coincided with the flu-prone season. Therefore, the seasonal surge and abnormality of non-COVID-19-related influenza symptoms may overlap with the early symptoms of COVID-19 infection. These factors may affect the public's judgment of influenza and COVID-19. In order to better understand the public's interest in "influenza" during the COVID-19 pandemic, we conducted Baidu Index Research to analyze the public's internet search for "influenza" from January 1, 2018, to January 1, 2022.

## Materials and methods

As a data platform based on the behavior and interest of a large number of internet users, one of the important functions of the Baidu Index is to retrieve the search trend and geographical pattern differences of keywords, so as to deeply understand the interest and behavior of Internet users. In fact, the personal computer (PC) trend of the Baidu Index has accumulated data since June 2006, and the data on mobile search trends has been available since January 2011. 

In order to understand the public's social search behavior and interest in "influenza", we used Baidu Index as a research tool, and generated the Baidu Index of "influenza" and "COVID-19" from January 1, 2018, to January 1, 2022. It is worth noting that the two expressions flu and influenza in English only correspond to the word "流感" in Chinese. Therefore, flu and influenza are used interchangeably.

Based on the average value of the search index in mainland China from 2018 to 2021, this study conducted a global spatial autocorrelation analysis using ArcGIS version 10.4 software (https://www.esri.com/). Spatial autocorrelation is a fundamental concept in spatial analysis [12]. It is the correlation among values of a single variable strictly attributable to their relatively close locational positions on a two-dimensional surface, introducing a deviation from the independent observation assumption of classical statistics [13]. Spatial autocorrelation was employed in this study. Depending on the content of the analysis, the spatial autocorrelation analysis can be divided into global spatial autocorrelation and local spatial autocorrelation. Global spatial autocorrelation is mainly used to analyze the spatial distribution and aggregation characteristics of the studied variables at certain significant levels.

**Figure 1:**
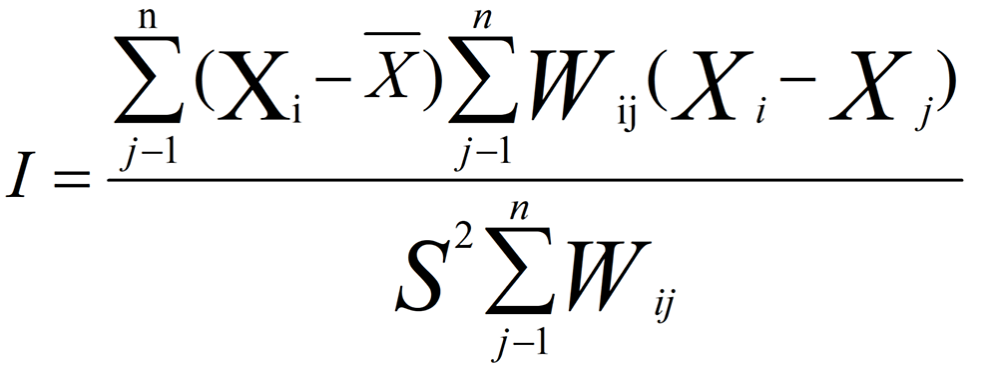
The Global Moran'I index

In this study, the Global Moran'I index was used to analyze whether there is spatial clustering of public concern about "influenza" in 31 provinces (municipalities directly under the central government and autonomous regions) in China, except Hong Kong, Macao, and Taiwan. The specific calculation formula is shown in Figure 1.

In the mathematical equation (as shown in Figure 1), I is the Global Moran's I index, n is the number of evaluation units, X_i_ and X_j_ are the total annual "influenza" concerns of 31 provinces (municipalities directly under the Central Government and autonomous regions), and W_ij_ is the weight matrix of a unit and its neighboring units. I is the Moran index, which is mainly used to evaluate the spatial aggregation characteristics of the observed indicators, and its value ranges from -1 to 1. When I is equal to 0, it means that the spatial distribution of the observed indicators is randomly distributed, and there is no spatial autocorrelation; when I is greater than 0, it means positive correlation, and the observed indicators show a spatial aggregation pattern; when I is less than 0, it means negative correlation, and the observed indicators show a discrete pattern in space.

**Figure 2:**
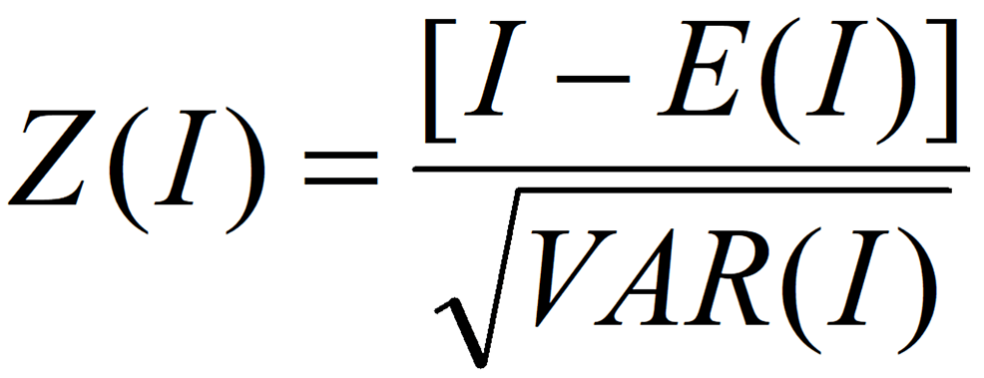
Z-Score

In order to ensure the correctness of Global Moran's I index, Z-Score is usually used to test the significance of the index to ensure the accuracy of the results. The calculation formula is shown in Figure 2.

In a mathematical equation (as shown in Figure 2), Z(I) denotes the significant level, E(I) is the expectation of I value, VAR(I) is the expectation of the I value, and VAR(I) is the variance of I value, and a 95% confidence interval is taken for significance testing, i.e., the critical value is 1.96. When |Z| is greater than 1.96, it indicates the existence of significant spatial autocorrelation, and when |Z| is less than 1.96, it indicates a random distribution of the observed values. When Z is greater than 1.96, there is a positive correlation with a clustered distribution, and when Z is less than -1.96, there is a negative correlation with a discrete distribution.

Local spatial autocorrelation is mainly used to analyze the specific spatial geographic distribution location of aggregation sites. Since Moran's I index does not reflect the correlation degree between each province (municipalities directly under the central government and autonomous regions) and its surrounding, local spatial autocorrelation is introduced for analysis. The local spatial autocorrelation can explore the spatial information of the aggregation location of the observations in space, reflecting the correlation degree of the observations with their critical units in a certain spatial unit.

**Figure 3:**
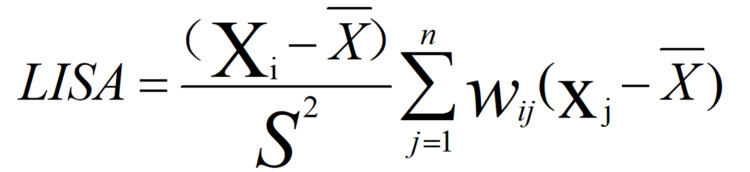
LISA mathematical equation LISA: Local indices of spatial association

**Figure 4:**
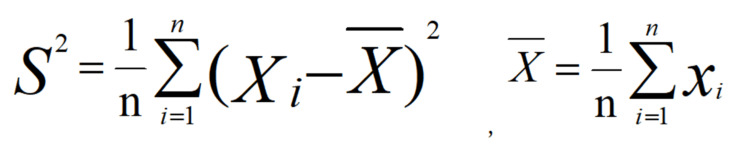
Variance and mean

In the mathematical equation shown in Figure 3, w_ij_ is the spatial weight matrix, x_i_ and x_j_ are the attention degree of spatial units. In the mathematical equation shown in Figure 4, X and S^2^ are the mean and variance of public attention, respectively. According to the magnitude of the local Moran index, the spatial aggregation attributes can be classified into four categories: high - high (H-H), high - low (H-L), low - low (L-L), and low -high (L-H). Among them, "high-high" and "low-low" indicate the spatial aggregation of spatial attention, while high-low and low-high indicate the spatial dispersion or variability of public attention.

## Results

**Figure 5:**
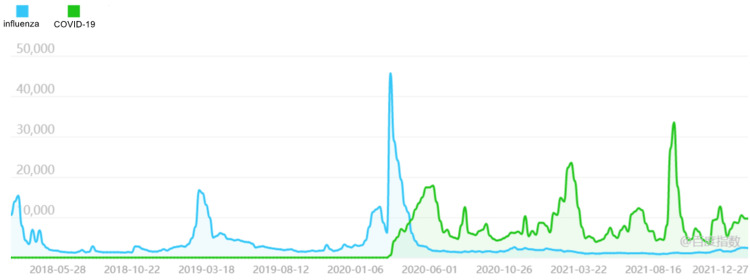
Baidu index of "influenza" in China (Y-axis means search index)

We observed that before the outbreak of COVID-19, the search trend of the public for "influenza" was concentrated in winter and spring, showing seasonal characteristics. After the outbreak of COVID-19, the search for "influenza" increased significantly, reached a peak that had never been seen before, and then rapidly decreased to a lower level, but no longer showed the characteristics of seasonal changes (as shown in Figure 5). This shows that there is an important connection between the COVID-19 and the online search for "influenza".

The results are shown in Table 1, which shows that the Global Moran's I index of public concern about "influenza" from 2018 to 2021 shows an overall increasing trend, with a decrease of 0.1190 in 2019 and a maximum of 0.1577 in 2021. Although the value of Global Moran's I index is smaller, it is still greater than 0. Therefore, the concern about "influenza" in China is spatially clustered. During the COVID-19 epidemic, the spatial correlation between the magnitude of public concern and the spatial correlation becomes larger as the number of years increases and is greater than that before the COVID-19 outbreak.

**Table 1:** Moran's I index of public concern for "influenza" in 2018-2021

Year	2018	2019	2020	2021
Moran’s I	0.1479	0.1190	0.1546	0.1577

In this study, we used Arc GIS version 10.4 software to conduct a local spatial autocorrelation analysis of public concern about "influenza" in 31 provinces (municipalities directly under the Central Government and autonomous regions) in China in 2018 and 2021, except Hong Kong, Macao, and Taiwan and selected Local indices of spatial association (LISA) aggregation diagrams for the analysis, and the results are shown in Table 2. As shown in Table 2, in 2018, i.e., the year before the new pandemic, there were three provinces and cities with positive high-high local spatial autocorrelation type, accounting for 9.7% of the country, namely, Shanghai, Jiangsu, and Zhejiang, and two provinces and cities with positive low and low types, accounting for 6.5% of the country, namely, Qinghai and Tibet. There are four provinces and cities with positive high-high correlation type, accounting for 12.9% of the country, namely, Jiangsu, Zhejiang, Shandong, and Henan; there are two provinces and cities with positive low and low correlation type, accounting for 6.5% of the country, namely, Qinghai and Tibet. In terms of local spatial distribution, compared with the pre-new crown, the spatial aggregation of areas during the new crown pandemic showed an expanding trend, indicating that the public concern about "influenza" in Shanghai has decreased, while the public concern of "influenza" in Shandong and Henan has increased. The main types of local spatial autocorrelation in 2018 and 2021 are both positive high-high correlations, but the former is mainly concentrated in the eastern coastal region, while the latter begins to spread to the central region.

**Table 2:** Types of local spatial autocorrelation based on the public concern of "influenza" in 2018-2021

Year	High-High	High-Low	Low-Low	Low-High
2018	Shanghai, Jiangsu, Zhejiang	—	Qinghai, Tibet	—
2021	Jiangsu, Zhejiang, Shandong, Henan	—	Qinghai, Tibet	—

## Discussion

Even though there are multiple types of research about influenza previously through Google Trends, this Baidu Index study is the first time to explore the public's interest and behavior in "influenza" before and after COVID-19. The study showed that after the outbreak of COVID-19, the public search index for "influenza" suddenly rose, then fell, and remained at a low level. It is worth noting that during the COVID-19, the "flu" Baidu search trend no longer showed the seasonal change characteristics before the new disease outbreak. This suggests that there is an important relationship between COVID-19 and the online search for "influenza".

At the beginning of the outbreak of COVID-19, the search volume for "influenza" increased rapidly, reaching an unprecedented peak. Therefore, we speculate that the outbreak time of the epidemic is the season of frequent influenza, and the symptoms of patients with new coronavirus are like influenza [7-11]. It is difficult for the public to distinguish between the two, coupled with the fear of the epidemic, which has led to a surge in a public search for "influenza".

During the period of the COVID-19 pandemic, the search volume of the public for "influenza" has been kept at a low level and no longer presents the characteristics of seasonal changes. In addition, according to relevant papers, during the epidemic period of COVID-19, public health interventions significantly reduced seasonal influenza [14-18]. This has led to a hypothesis that COVID-19 has started the response to major public health emergencies and implemented a variety of intervention measures. While preventing the spread of COVID-19, it has also hindered the spread of the influenza virus, resulting in a significant reduction in influenza activities. Therefore, during the COVID-19 pandemic, due to the reduction in the number of patients, the public's online search for "influenza" has been maintained at a low level. So, it could be concluded that public health interventions can significantly reduce seasonal influenza activities and can be used as effective strategies and measures to prevent and control seasonal influenza and other infectious diseases in the future.

While viral competition and increased vaccination may have contributed, we propose that the most influential process in suppressing the influenza epidemic of 2020-2021 were the significant behavioral interventions in place due to the COVID-19 pandemic. Nonpharmaceutical interventions such as closures of schools and non-essential businesses, telework, restriction on gathering size [11], and mask-wearing have been key public health tools for limiting the impact of the COVID-19 pandemic [19,20].

Significantly, this study used Arc GIS version 10.4 software to conduct spatial autocorrelation analysis on the average values of search indices of 31 provinces (municipalities and autonomous regions) in China, excluding Hong Kong, Macao, and Taiwan, from 2018 to 2021. It is noted that the spatial autocorrelation analysis includes Global Moran's I and Local Moran's I.

For Global Moran's I, during the COVID-19 pandemic, the magnitude of public concern became more and more spatially correlated as the year increased and was greater than before the COVID-19 outbreak. The reason for this is mainly that the number of patients with COVID-19 and presenting provinces are updated daily during the COVID-19 pandemic, and the clinical presentation of COVID-19 has a significant similarity to influenza [4]. This would prompt the public in the provinces where the patients with COVID-19 are located to search the Internet for information related to COVID-19 along with information on influenza.

For Local Moran's I, the high local aggregation of "influenza" concerns in the central and eastern regions may be due to the fact that both influenza and 2019 coronavirus disease are infectious respiratory diseases [5], and the economically developed central and eastern regions of China have frequent human and logistic exchanges, which contribute to the spread of the diseases.

At the same time, some limitations related to this analysis need to be considered. These searches may not represent all cases from China, mainly due to limited access to Internet services in some regions. In addition, our analysis only refers to Baidu Index and does not consider other search platforms. In this analysis, there are some speculative observations due to the lack of detailed patient-level data, i.e. no detailed history of influenza. However, our analysis serves as a good preliminary source to provide a wide range of population views on "influenza" in the pre-and post-pandemic period of COVID-19.

We encourage further research to find answers to the following questions. (a) When COVID-19 first broke out, the public's search for "influenza" soared. Was it because of the panic caused by the outbreak or other reasons?; (b) Will the COVID-19 pandemic prevent people from going to medical institutions to search for symptoms or treatments online?; (c) After the COVID-19 is well controlled, will the public's influenza search index return to the seasonal trend before the new crown?; (d) Are public safety interventions taken during the COVID-19 epidemic effective in preventing and reducing the spread of other infectious diseases?; (e) What experience can be used for reference for the intervention and prevention and control of infectious diseases in the future? Will the public's relevant health and safety awareness be strengthened?

In conclusion, during the COVID-19 pandemic, the trend of online search for "influenza" increased first and then decreased, and remained at a low level. This phenomenon prompted people to think about the effectiveness of public safety interventions in preventing and stopping infectious diseases.

## Conclusions

The analysis of the Baidu Index shows that before COVID-19, the public search for "influenza" showed the characteristics of seasonal changes. However, during the COVID-19 pandemic, the "influenza" search trend first soared and reached a peak. After that, it decreased sharply and remained at a low level, and there was no seasonal change. This suggests that there may be a relationship between COVID-19 and influenza. We speculate that the public intervention measures taken during the COVID-19 pandemic may be one of the main reasons for the reduction of influenza cases. The public health intervention measures taken during the epidemic not only slowed down the spread of the epidemic but also prevented the spread of seasonal infectious disease influenza, which led to a low level of "influenza" search trend during the new coronavirus pandemic. In addition, the spatial clustering of "influenza" concerns across China, as well as the increasing spatial correlation of public concerns, is greater than before the COVID-19 outbreak. This is related to the daily update of information on the number of patients suffering from COVID-19 and the emergence of provinces. Meanwhile, the "high-high" local clustering of "influenza" concerns in the central and eastern regions of China during the COVID-19 pandemic may be due to the frequent population movement and logistics in the central and eastern regions, which contributed to the spread of influenza and COVID-19 epidemics. It is possible that the frequent population movement and logistics in the central and eastern regions have contributed to the spread of influenza and the COVID-19 pandemic.

Baidu Index can prove to be a convenient tool for real-time monitoring of influenza, COVID-19, and other infectious diseases, as well as the change of public interest in "influenza", which also reflects the effectiveness of public health measures in intervening and preventing infectious diseases.
